# Factors influencing treatment outcome in patients with gastroesophageal reflux disease: outcome of a prospective pragmatic trial in Asian patients

**DOI:** 10.1186/1471-230X-14-156

**Published:** 2014-09-09

**Authors:** Khean Lee Goh, Kee Don Choi, Myung-Gyu Choi, Tsai-Yuan Hsieh, Hwoon-Yong Jung, Han-Chung Lien, Jayaram Menon, Steven Mesenas, Hyojin Park, Bor-Shyang Sheu, Justin CY Wu

**Affiliations:** University of Malaya, Kuala Lumpur, Malaysia; Asan Medical Center, University of Ulsan College of Medicine, Seoul, Korea; Seoul St Mary’s Hospital, Seoul, Korea; Tri-Service General Hospital, Taipei, Republic of China; Taichung Veterans General Hospital, Taichung, Republic of China; Queen Elizabeth Hospital, Kota Kinabalu, Malaysia; Singapore General Hospital, Singapore, Singapore; Gangnam Severance Hospital, Seoul, Korea; National Cheng Kung University Hospital, Tainan, Republic of China; The Chinese University of Hong Kong, Shatin, Hong Kong SAR, Republic of China

## Abstract

**Background:**

Predicting response to proton pump inhibitor (PPI) treatment can aid the effective management of gastroesophageal reflux disease (GERD). The aim was to investigate the predictors of symptomatic response to pantoprazole in Asian patients with GERD; the first study of its kind in Asian patients.

**Methods:**

Asian patients with GERD symptoms (N = 209) received pantoprazole 40 mg daily for 8 weeks in a multinational, prospective, open-label study. Response was assessed using ReQuest™. Baseline and demographic factors were examined using logistic regression to determine if they were related to treatment response.

**Results:**

Response rates were 44.3% (Week 4) and 63.6% (Week 8) in Asian patients versus 60.7% (*P* < 0.001) and 72.2% (*P* = 0.010) for the rest of the world. Higher response rates at 8 weeks occurred in patients with erosive reflux disease (ERD; 71.3%) versus those with non-erosive reflux disease (NERD) at baseline (48.5%). The presence of ERD (*P* = 0.0143) and lower ReQuest™-GI scores at baseline (*P* = 0.0222) were associated with response. Improvements in quality of life (QoL) and anxiety and depression at 4 and 8 weeks were associated with treatment response (both *P* < 0.0001). Patient satisfaction correlated with treatment response (*P* < 0.0001), and improvement in anxiety and depression (*P* < 0.0001) and QoL (*P* < 0.0001).

**Conclusions:**

Asian patients with GERD, especially those with NERD, may have lower response rates to PPIs than Western populations. ERD and less severe gastrointestinal symptoms may help to predict symptomatic responses to PPIs in Asian patients.

**Trial Registration:**

ClinicalTrial.gov identifier: NCT00312806.

## Background

Despite the potency of proton pump inhibitors (PPIs) on gastric acid secretion, many patients with gastroesophageal reflux disease (GERD) continue to experience reflux symptoms while receiving PPI therapy [1–3]. Thus, in clinical practice, it is important for physicians to understand whether a patient with GERD will respond well or poorly to the prescribed treatment. To address this question, a large worldwide study in GERD patients designed to approximate ordinary clinical practice was undertaken. The study aimed to identify patient characteristics predicting symptomatic response to pantoprazole treatment (results for the total population have been previously published [4]).

Although GERD is less common in Asian populations than in Caucasian populations, the prevalence has been increasing and it has become an important disease in the region [5–8]. Endoscopy-based studies tend to show a prevalence of erosive esophagitis of >10% and symptom-based studies show a prevalence of 6–10% [5]. As with other populations, a substantial portion of Asian patients with GERD fail to respond to PPI therapy. In one study, 20% of patients with erosive reflux disease (ERD) and 33% of those with non-erosive reflux disease (NERD) did not achieve adequate response after receiving a PPI [9]. Here we report an analysis of data from five countries – Hong Kong, Korea, Malaysia, Singapore and Taiwan – to investigate the predictors of symptomatic response to pantoprazole in Asian patients with GERD. This is the first study of its kind performed in Asian patients.

## Methods

### Study design

Asian patients were enrolled as part of a large multicenter, multinational, prospective, open study conducted from May 2006 to March 2007 (ClinicalTrial.gov identifier: NCT00312806), the full trial design details of which have been previously reported [4]. In brief, a pragmatic study design was adopted to resemble conditions of ordinary clinical practice as much as possible, thereby optimizing the likelihood that the results would be relevant to everyday practice [10, 11]. The study was developed in accordance with the Declaration of Helsinki and ethics approval was obtained from the relevant local ethics committee: Joint Chinese University of Hong Kong- New Territories East Cluster Clinical Research Ethics Committee (Hong Kong); Medical Research & Ethics Committee, Institute of Medical Research (Malaysia); Medical Ethics Committee, University Malaya Medical Center (Malaysia) Singapore General Hospital Institutional Review Board (Singapore); Institutional Review Board Asan Medical Center (South Korea); Institutional Review Board, The Catholic University of Korea, Kangnam St. Mary’s Hospital (South Korea); Institutional Review Board, Seoul National University Hospital (South Korea); Institutional Review Board, Yongdong Severance Hospital (South Korea); Institutional Review Board, Samsung Medical Center (South Korea); Human Experiment and Ethics Committee, National Cheng Kung University Hospital (Taiwan); Joint Institutional Review Board (Taiwan); Institutional Review Board of Tri-Service General Hospital, National Defense Medical Center (Taiwan). All patients provided written informed consent prior to enrolment.

Eligible patients were aged ≥18 years and had symptoms considered by the investigating physician to justify a diagnosis of GERD, without further specification of GERD diagnostic criteria. Patients were excluded from the study if they had symptoms or evidence of complicated GERD, previous upper gastrointestinal (GI) surgery or had received *Helicobacter pylori* (*H. pylori*) eradication treatment in the 4 weeks preceding the study. Those who had recently taken acid-suppressing medications, corticosteroids, non-steroidal anti-inflammatory drugs (NSAIDs) or prokinetics were also excluded. During the study, patients were not permitted to use acid-suppressing medications, corticosteroids, NSAIDs, prokinetics, sucralfate, misoprostol, bismuth preparations, substances that affect the relief of acid-related symptoms, ketoconazole or drugs showing pH-dependent absorption.

After enrolment, upper GI endoscopy was performed. Patients were categorized as having ERD or NERD, and grade of esophagitis was assigned according to the Los Angeles classification [12, 13]. Patients with esophageal stricture, a Schatzki’s ring, an esophageal diverticulum, esophageal varices, or Barrett’s esophagus on endoscopy were excluded. At this time, *H. pylori* status was determined by serology [14]. Participants received pantoprazole 40 mg to be taken once daily before breakfast over the 8-week study period. Patients were required to attend the investigation center on three occasions during this period.

### Assessments

#### ReQuest™ questionnaire

ReQuest™ is a self-administered questionnaire that assesses seven dimensions of GERD (acid complaints, upper abdominal/stomach complaints, lower abdominal/digestive complaints, nausea, sleep disturbances, other complaints, and general well-being) to provide a comprehensive evaluation of the intensity and frequency of symptoms [15–17]. The dimensions of ReQuest™ can be grouped into two sub-scales: ReQuest™-GI, which includes acid complaints, upper abdominal/stomach complaints, lower abdominal/digestive complaints, and nausea, and ReQuest™-WSO, which is comprised of general well-being, sleep disorders, and other complaints. Both a long and a short version of ReQuest™ have been validated in several languages. The short version of ReQuest™ was used in this study, and was completed by participants on Day 0 (i.e. the day before commencing treatment) and then daily thereafter. Patients were determined to be treatment ‘responders’ if their ReQuest™-GI symptom score was below 1.6 on three consecutive days. A score of 1.6 was determined as the threshold symptom score for ReQuest™-GI in an international study of 1167 healthy subjects who completed ReQuest™ on four consecutive days. The intensity and frequency of ReQuest™ dimensions were scored and weighted and the sum scores of ReQuest™ and its subscales corresponding to the 95% percentiles were calculated as threshold scores [18]. These scores corresponded with those obtained for a German population [19], indicating that the threshold concept was reliable and valid for use in clinical trials [18, 19].

#### HADS, GERDyzer™ and treatment satisfaction

The Hospital Anxiety Depression Scale (HADS) and GERD Analyzer (GERDyzer™) questionnaires were completed on Day 0. These questionnaires, along with the treatment satisfaction sheet, were then completed at Week 4 and Week 8. The treatment satisfaction sheet was used to categorize patient satisfaction with symptom control during the 24 hours preceding the Week 4 and Week 8 study visit as ‘very satisfied’, ‘fairly satisfied’ or ‘not satisfied’. The HADS is a well-established screening measure for anxiety and depression used in outpatient clinics. In this study, the HADS was used to assess relationships between the patients’ symptoms and psychological constitution, according to the standard scoring method [20, 21].

Finally, the GERDyzer™ is a questionnaire that evaluates the impact of GERD on a patient’s quality of life across 10 dimensions: general well-being, pain/discomfort, physical health, energy, daily activities, leisure activities, social life, diet/eating/drinking habits, mood and sleep [22]. Each dimension is assessed using a 100 mm visual analogue scale ranging from ‘not at all’ to ‘very much’, with the exception of ‘general well-being’, which is assessed from ‘excellent to ‘unbearably bad’. Higher GERDyzer™ scores indicate greater impairment of quality of life.

### Statistical analysis

All efficacy data are presented for the intent-to-treat (ITT) population (N = 209), which consists of all patients who received at least one dose of the study medication. Safety data are presented for 210 patients who were recruited in the study. Age, body mass index (BMI), gender, smoking status, *H. pylori* status, symptoms suggesting irritable bowel syndrome, presence of esophagitis before treatment, baseline ReQuest™-GI score, and the HADS total and sub-scores were investigated as possible influences on response to treatment at 8 weeks using applicable statistical tests (two-group t-tests for numerical data and the chi-square test for categorical data). A p-value of <0.1 was taken to indicate significant influence of the independent variable. Following univariate analysis, a global multivariate logistic regression was performed using the response to treatment of patients as the dependent variable and those variables identified as possibly having an influence on response as independent variables, to take into account any effects of confounding. Results were not analyzed at the individual country level because of small sample sizes.

A possible association between GERDyzer™ score and the HADS total score was examined for each visit using Pearson’s correlation coefficient. Similarly, an association between GERDyzer™ score and patient satisfaction, and the HADS total score and patient satisfaction, was examined for each visit under treatment, and the post-treatment patient satisfaction and baseline scores were examined using Pearson’s correlation coefficient.

## Results

In total, 210 participants were recruited in five Asian countries; this comprised the safety population. The ITT population comprised 209 individuals. Baseline and demographic characteristics are presented in Table 1.

**Table 1:** **Baseline and demographic characteristics**

Gender, n (%)	
Male	101 (48.3)
Female	108 (51.7)
Age, years, mean (SD)	48.5 (12.12)
Height, cm, mean (SD)	164.1 (7.97)
Weight, Kg, mean (SD)	64.9 (12.20)
BMI, mean (SD)	24.0 (3.50)
Cigarette smoking, n (%)	
Never	150 (71.8)
Former	27 (12.9)
Current	32 (15.3)
Country of origin, n (%)	
Hong Kong	26 (12.4)
South Korea	86 (41.1)
Malaysia	38 (18.2)
Singapore	7 (3.3)
Taiwan	52 (24.9)

Intent-to-treat population (N = 209).

*BMI*, Body mass index; *SD*, Standard deviation.

### Response rates

**Figure 1:**
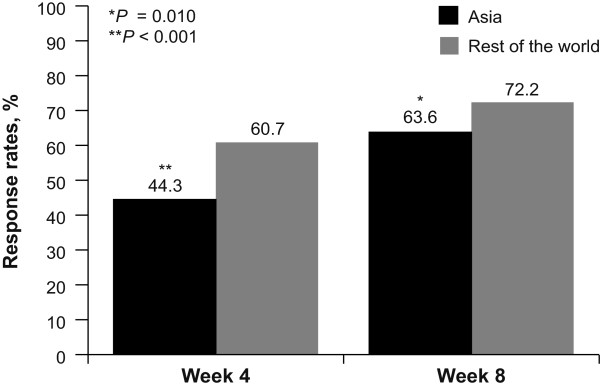
**Percentage of patients responding to pantoprazole 40 mg once daily at Week 4 and Week 8 of treatment.** A comparison of Asian populations (combined: Hong Kong, Korea, Malaysia, Singapore and Taiwan) with the rest of the world.

The overall response rates (i.e., ReQuest™-GI symptom score below 1.6 on 3 consecutive days) for the ITT population were 44.3% at Week 4 and 63.6% at Week 8 (Figure 1). This was lower than those reported for the rest of the world (16 countries: Europe, UK, North and South America, Africa, India and Australia) (60.7 at Week 4 [*P* < 0.001, chi square] and 72.2 at Week 8 [*P* = 0.010, chi square]). At the end of the 8 weeks of treatment, patients who had ERD pre-treatment showed higher response rates than those with NERD (71.3% vs. 48.5%). Response rates for patients with baseline ERD and NERD in Asia follow a similar pattern to those seen in the rest of the world (69.6% and 60.7%), although there tends to be fewer responders in the NERD group.

In univariate analyses other variables studied, including age, gender, cigarette smoking and *H. pylori* status, had no significant effect on response to pantoprazole. HADS scores were the exception, with lower mean baseline HADS total scores (*P* = 0.0149) and lower mean baseline depression subscale scores (*P* < 0.0128) occurring in responders versus non-responders.

Multiple logistic regression analysis data assessing the influence of various factors on response rates following pantoprazole treatment are presented in Table 2. The presence of ERD (*P* = 0.0143) and lower ReQuest™-GI scores at baseline (*P* = 0.0222) were associated with a response to therapy. BMI and HADS subscores had no significant influence on response rates.

**Table 2:** **Factors influencing treatment response**

Factor	Logistic regression ***P***-value
ERD/NERD	0.0143
ReQuest™-GI at baseline	0.0222
BMI	0.0831
HADS	
Anxiety subscale	0.7355
Depression subscale	0.2130

*BMI*, Body mass index; *ERD*, Erosive reflux disease; *GI*, Gastrointestinal; *HADS*, Hospital Anxiety and Depression Scale; *NERD*, Non-erosive reflux disease.

**Figure 2:**
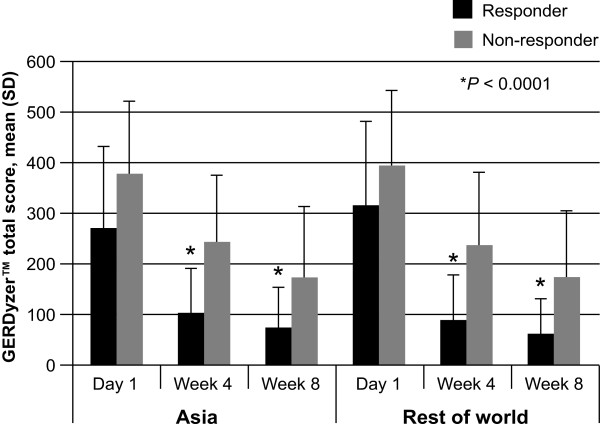
**Improvement in quality of life (total GERDyzer** ^**TM**^ **score) by patient response to treatment (assessed using ReQuest** ^**TM**^ **-GI).** A comparison of Asian populations (combined: Hong Kong, Korea, Malaysia, Singapore and Taiwan) with the rest of the world.

**Figure 3:**
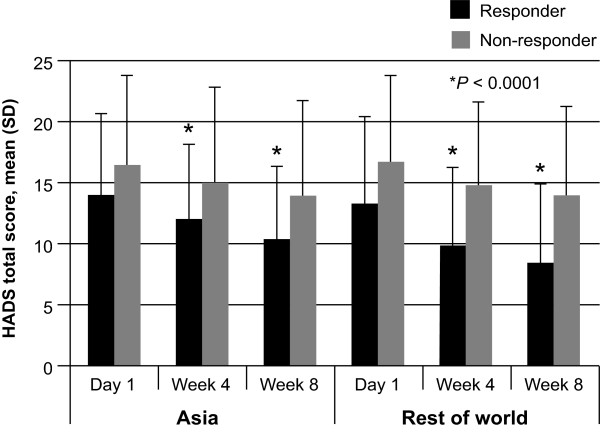
**Improvement in anxiety and depression (HADS score) by patient response to treatment (assessed using ReQuest** ^**TM**^ **-GI).** A comparison of Asian populations (combined: Hong Kong, Korea, Malaysia, Singapore and Taiwan) with the rest of the world.

Improvement in quality of life (as assessed by GERDyzer™ scores) at 4 and 8 weeks, was associated with response to treatment, with responders in the Asian population scoring lower on GERDyzer™ than non-responders at each time point (Figure 2; *P* < 0.0001). Similar results were observed for the rest of the world (Figure 2; *P* < 0.0001). In addition, response to treatment was associated with improvement in anxiety and depression, as indicated by a lower HADS total score, at 4 and 8 weeks in both the Asian population and the rest of the world (Figure 3; both *P* < 0.0001).

### Control of symptoms and patient satisfaction

**Figure 4:**
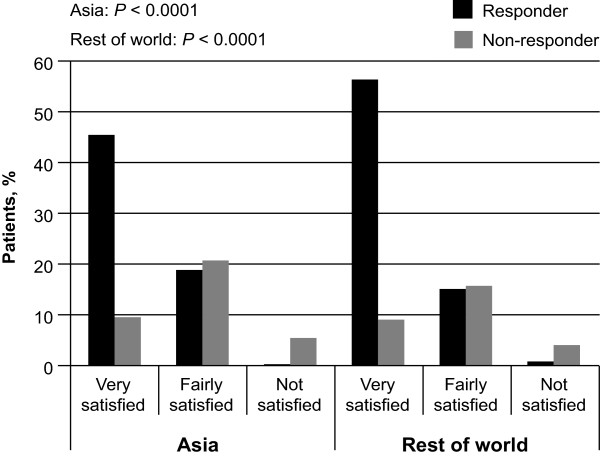
**Proportion of responders versus non-responders satisfied with symptom control in the preceding 24 hours.** A comparison of Asian populations (combined: Hong Kong, Korea, Malaysia, Singapore and Taiwan) with the rest of the world.

At 8 weeks following treatment, more patients who had responded to treatment than had not responded reported that they were satisfied with symptom control in the preceding 24 hours in both the Asia population (*P* < 0.0001) and the rest of the world (*P* < 0.0001; Figure 4). Furthermore, greater satisfaction with treatment at Week 4 and Week 8 was reported in those who had improvements in anxiety and depression (assessed by total HADS score) at these same time points (Week 4: *P* < 0.0001; Week 8: *P* < 0.0001), irrespective of baseline HADS scores. Similarly, improved quality of life (assessed by GERDyzer™) was also associated with greater patient satisfaction at Week 4 (*P* < 0.0001) and Week 8 (*P* < 0.0001), irrespective of GERDyzer™ scores at baseline.

### Safety

Over the course of the study, 74 adverse events (AEs) were reported by 50 patients in the safety set (23.8%), a similar rate to that reported for the rest of the world (25.2% of 1718 patients). Most AEs were of mild intensity (86.5%) and were considered unrelated to the study medication by the investigator (77.0%). Only 5.4% of AEs were considered to be likely related and none were considered to be definitely related to the study medication. Two patients (1%) experienced treatment-emergent serious adverse events (SAE) during the trial. Both SAE’s were of severe intensity. One patient had myalgia, which was assessed as unlikely related to the study medication by the investigator. The sudden hearing loss of the other patient was assessed to have no relation to the study medication by the investigator. No patients died during the study.

The most common treatment-emergent AEs were diarrhea (5.2%), upper respiratory tract infection (3.3%) and nasopharyngitis (2.9%); other events occurred in <2% of the population (Table 3). Treatment-emergent AEs in the Asian population generally occurred at a similar frequency to those in participants from the rest of the world, with the exception of diarrhea, which was more commonly reported in Asia (5.2% vs. 2.7%), and headache, which was more commonly reported in the rest of the world (1.4% vs. 4.0% for Asia vs. the rest of the world).

**Table 3:** **Frequently reported treatment-emergent adverse events**

MedDRA preferred term	Asia (N = 210), n (%)	Rest of the world (N = 1718), n (%)
Diarrhea	11 (5.2)	47 (2.7)
Upper respiratory tract infection	7 (3.3)	8 (0.5)
Nasopharyngitis	6 (2.9)	10 (0.6)
Headache	3 (1.4)	69 (4.0)
Vomiting	3 (1.4)	10 (0.6)
Constipation	2 (1.0)	18 (1.0)
Heart rate increased	2 (1.0)	0 (0.0)
Insomnia	2 (1.0)	13 (0.8)
Nausea	0 (0.0)	33 (1.9)
Abdominal pain	1 (0.5)	22 (1.3)
Influenza	0 (0.0)	19 (1.1)

A comparison of Asian populations with the rest of the world (intent-to-treat population, N = 210).

## Discussion

This is the first international, multicenter outcome study in patients with GERD in Asia. In the setting of everyday clinical practice, response rates to pantoprazole treatment at 8 weeks were 63.6% across five Asian populations, indicating that some patients fail to adequately respond to PPI therapy. Two features, the presence of erosive disease and less severe GI symptoms, seemed predictive of response and these may be helpful in determining the likely success of pantoprazole treatment in controlling symptoms in GERD in Asian patients, thereby helping to manage patient expectations. More complete symptom control, as assessed by ReQuest™-GI, was associated with greater patient satisfaction, better quality of life, and greater improvements in anxiety and depression, at Weeks 4 and 8.

This analysis was part of a larger study designed to obtain information relevant to everyday clinical practice, whereby patients with GERD often present with a symptom burden that is more complex than heartburn alone [23], many patients do not have esophagitis and treatment with a PPI is undertaken without first obtaining an upper GI endoscopy. It should be noted that pantoprazole 20 mg is indicated in patients with symptomatic GERD [24]. However, to replicate clinical practice where treatment is usually started on the basis of clinical history rather than endoscopy, a 40 mg dose of pantoprazole was chosen for all patients in this study. The higher dosage used in NERD patients could have potentially increased response rates, although the impact of this is likely to be small as previous studies have shown similar symptom response rates with pantoprazole 20 and 40 mg, even in patients with mild GERD [25]. The study had a pragmatic trial design and patients were enrolled in the trial on the basis of a clinical history of GERD, which allowed for the collection of data relevant to daily clinical practice, unlike trials of explanatory design, which may enroll a patient population that is not representative of that being treated in ordinary practice [10, 11]. Explanatory trials often overestimate the success of treatment compared with outcomes achieved in ordinary clinical practice [26].

The current analysis provides a treatment response rate of 63.6% (Week 8) across Asia, which is slightly lower than those previously reported in the literature [9]. PPI response rates were 80% in patients with ERD and 67% in those with NERD in one study [9]. Of note, these response rates are also lower than those reported for the rest of the world, although low response rates were also observed in Australia in the previously published analysis, in which the influence of geography on response rates was assessed. These overall results for the Asian population may be driven by the lower response rates in patients with NERD (48.5%) than in those with ERD (71.3%), as these vary substantially from data for the rest of the world (60.7%). This is consistent with other studies that have shown that patients with NERD tend to respond less well to PPI treatment than those with ERD [1, 27, 28]. While Asian patients were included in the international validation studies for both ReQuest™ and GERDyzer™, it has also been reported that the interpretation and reporting of reflux symptoms is subject to ethnic variation [29], with Asian populations experiencing more atypical symptoms, such as chest pain, and Western populations reporting more heartburn [5, 6]. Of consideration, there is lack of appropriate terminology in some Asian languages for heartburn [30] and one study has shown that patients of East Asian origin did not understand the symptom of heartburn [29]. We cannot definitively rule out ethnic variation in symptom reporting as a possible cause for the differences between Asian populations and the rest of the world. Nonetheless the short version of ReQuest™ quantifies the four dimensions that make up ReQuest™-GI each on an individual 7-point Likert scale, without the mention to specific symptoms, suggesting that patients assess these scales based on their predominant symptoms [23]. Indeed, ReQuest™ was designed to avoid an assessment of symptom response based primarily on heartburn [23]. It should be noted that this sample is only reflective of the whole Asian population (as is the sample for the rest of the world). Obtaining a truly representative sampling across many countries is always difficult; nonetheless, data across as broad a population as possible were carefully collected in this multicenter study. Further studies on the ethnic differences in morbidity and treatment response of GERD patients in Asia are needed.

Patients with less severe GI symptoms (lower scores on ReQuest™-GI) showed a better response to pantoprazole treatment. Previous reports have shown that treatment outcome may be predicted by symptom severity before or shortly after commencing treatment [31–35]. Previously reported results for the total population suggest that ReQuest™ scores at baseline have some predictive ability for outcomes at Week 8, although the authors suggested that the predictive accuracy may be lower than physicians would wish [4]. Nonetheless, the scores obtained from the abbreviated form of ReQuest™ (ReQuest in Practice™ [36]) have been shown to be more accurate than the physicians’ conventional clinical enquiry in identifying patients whose symptoms would continue to be controlled after stepping down from full dose to half dose PPI therapy [37], suggesting value in the systematic assessment of symptom burden in clinical practice.

It might be expected that patients with concurrent anxiety or depression would respond poorly to GERD treatment, as has been shown in previous studies [32, 38]. Although in the current study higher mean baseline HADS total scores and depression subscale scores predicted non-response to pantoprazole treatment in univariate analyses, this was not borne out in the multivariate analysis. However, improvement in anxiety and depression with treatment was associated with more complete symptoms control and greater patient satisfaction following 4–8 weeks’ treatment. This potentially supports the notion that psychological distress, such as anxiety and depression, may be a consequence of poorly controlled GERD symptoms, rather than a contributing factor to severe reflux symptoms. However, this study did not contain a placebo group; therefore, there is the potential that response to these subjective psychological measures may have been over estimated. Interpretations of the data thus require further substantiation.

Lower BMI has been associated with poorer treatment response to PPI therapy in previous studies in Western populations [39], and also in the total worldwide population for the current study [4]. In this analysis of five Asian populations, BMI fell just short of statistical significance (*P* = 0.0831), which suggests that there might be a possible effect of BMI on treatment outcome (beta risk). Further research is required to confirm whether or not BMI affects response to PPI therapy in Asian populations as it does in other groups. Along with BMI, female gender, anxiety and IBS were also factors predicting response in the total global population for this study [4] but not in the Asian subgroup.

## Conclusions

Asian patients with GERD, especially those with NERD, appear to have lower response rates to PPI treatment than Western populations. Some readily identifiable features, such as the presence of erosive disease and less severe GI symptoms, may help to predict symptomatic responses to a PPI in Asian patients, which may help in managing patient expectations.
